# In-depth morphological evaluation of tooth anatomic lengths with root canal configurations using cone beam computed tomography in North American population

**DOI:** 10.1590/1678-7757-2019-0103

**Published:** 2020-01-31

**Authors:** Varun KULKARNI, Onurcem DURUEL, Emel Tuğba ATAMAN-DURUEL, Melek Didem TÖZÜM, Salvador NARES, Tolga Fikret TÖZÜM

**Affiliations:** 1 University of Illinois at Chicago College of Dentistry ChicagoIL USA University of Illinois at Chicago, College of Dentistry, Chicago, IL, USA.; 2 Hacettepe University Faculty of Dentistry Ankara Turkey Hacettepe University, Faculty of Dentistry, Ankara, Turkey.

**Keywords:** Tooth anatomy, Cone beam computed tomography, Root canal morphology, Endodontics, Crown size, Root size

## Abstract

**Objective:**

This study aimed to assess the association between tooth size and root canal morphology by using CBCT analysis.

**Methodology:**

In this retrospective study, tooth anatomic lengths (crown and root lengths, buccolingual and mesiodistal dimensions) of 384 patients were assessed and correlated with Vertucci’s root canal morphology classification. Data was analyzed for gender-related differences using the independent sample t-test, ANOVA, and the Pearson’s correlation for a possible relation between anatomic lengths and canal morphology.

**Results:**

The maxillary first and second premolars showed a greater predilection for Type IV and II variants, respectively, while the mandibular first premolar showed a greater predilection for Type II canal system. The root canal system of the mandibular second premolar showed maximal diversity (47% Type I, 30% Type II, and 20% Type III). The dimensions were greater in men regardless of tooth type. The most significant relation (p<0.05) between the anatomic size and canal morphology was observed in the maxillary first premolars, followed by the mandibular canines (buccolingual dimension) and the lower second premolars (crown length). Negative correlations existed between the crown length and the patient’s age for the anterior teeth and mandibular second premolar (r=−0.2, p<0.01).

**Conclusions:**

The most common canal formation for anterior teeth was the Type I. The anatomic lengths had the strongest influence on the canal configuration of the maxillary first premolar, with Type IV being the most common root canal system. The mandibular second premolars showed maximal diversity in the canal classification terms and had a significant correlation with their crown lengths.

**Clinical Relevance:**

The complex relationship between the canal morphology and anatomic tooth sizes need meticulous awareness and recognition during endodontic procedures, in conjunction with the demographic variabilities.

## Introduction

Root canal morphology varies greatly from tooth to tooth and is not a single uniform canal in many cases, but it can be highly complex from orifice to apex. Tooth canal heterogeneities have been reported, including but not limited to: apical ramifications, loops, C-shaped canals, double “s-shaped” canal curvatures and accessory canals.^1^ One of the fundamental prerequisites for a successful endodontic treatment is a comprehensive knowledge about the anatomy of the tooth; typically, the root canal shape and its diversities. Undetected root canals are reported as the primary reason for endodontic retreatment in 42% of the cases. An investigation on root canal geometry reported that variations in canal conformations have much more influence on the changes occurring during canal preparation when compared with those due to instrumentation techniques, thereby reiterating the significance of canal anatomy.^2^ Considering the vast individual, genetic, and ethnic variations, it becomes particularly essential to understand and gauge the morphologic details of root canals.^3 , 4^ This can help in minimizing failure rates and ensure long-term prognosis of a tooth undergoing endodontic retreatment.

A study evaluating 1400 permanent teeth in Turkish population reported more than one canal in 22% of maxillary lateral incisors.^5^ Maxillary anterior teeth have been found to have a lower prevalence of extra roots and extra canals compared with mandibular anterior teeth. The estimated prevalence for a second canal is 11% for mandibular central incisors and from 7% to 11% for mandibular lateral incisors.^6 - 9^ Root canal treatment for mandibular incisors is deemed harder than that of molars, and it is as difficult as that of mandibular two-canal premolars because of their small dimension and the high prevalence of two canals. A study assessing the root anatomy of mandibular anterior teeth found two previously unidentified root canal types. The first variant consisted of two separate canals extending from the pulp chamber to the mid-root region, where the lingual canal was divided into two, followed by the joining of all three canal elements in the apical third as one canal. In their second category, a single canal from the pulp chamber was divided into two in the middle third of the root, which then rejoined to form one canal that split again, exiting as three separate canals with separate foramina.^10^ A possible association between crown size and the pervasiveness of bifid canals in mandibular incisors has been documented. Therefore, having a comprehensive knowledge of the pulpal anatomy is critical.

Likewise, the mandibular first premolars are equally difficult to treat endodontically, because of its wide canal morphology variety and difficulty in accessing the second canal, with an approximate 12% treatment failure rate. More so the lingual propensity of its crown and the angled separation of the secondary canal can hamper the detection of the second canal, both radiographically and via tactile examination. The incidence of mandibular premolars with the prevalence of more than one canal in the first and second premolars is 27.8% and 8.9%, respectively.^11^ An accurate interpretation of the crown and root morphology of such teeth is warranted for precise diagnostic radiography. In a case study, the cervical half of the root in mandibular premolars with more than one canal is often wider.^12^ The facio-lingual curvatures of the root canal system, which are not often visualized by two-dimensional radiographs, may make cleaning and shaping procedures more difficult. This requires a simpler, yet more accurate method to diagnose and visualize the root canal morphology. As cone beam computed tomography technique (CBCT) has an excellent resolution and capacity to visualize root anatomy in three dimensions and a much lower patient radiation dose compared with the multislice computed tomography, it is considered more precise in providing details about extra canals, apical deltas, and canal type than the digital radiography, with a highly strong correlation between data acquisition via CBCT in all spatial planes, for histology specimens.^13^ Its precision is comparable to the canal staining and clearing technique, which is considered the gold standard for gauging canal morphology, in addition to its utility in *in vivo* application.^14^ Very few studies have evaluated the root and canal morphology of the anterior and premolar teeth with CBCT to establish any possible correlation with tooth crown and root lengths. However, because of the small sample size of those studies, the findings cannot be generalized to larger populations.^14 , 15^ Thus, this study seeks to fill adequate knowledge voids regarding canal morphology, with the ultimate goal to increase the success of the endodontic treatment by decreasing procedural errors. This retrospective and cross-sectional study aims to establish reference data for normal tooth lengths (including crown and root lengths) and common root canal system for the maxillary and mandibular anterior teeth and premolars, and to correlate the relationship between anatomic lengths and canal morphology using CBCT analysis. This study also aimed to detect significant differences in CBCT measurements between demographic factors (gender and age), and tooth anatomic lengths and canal classification relationships.

## Methodology

### Study samples

This study was approved by the Institutional Review Board at University of Illinois at Chicago (2017-0968). The CBCT scans from adult patients who visited the Department of Periodontics, College of Dentistry at the University of Illinois at Chicago for a variety of dental/oral indications (e.g., impacted teeth, sinus examination, implant planning, etc.) between January 1st, 2017 and August 31, 2017 were acquired using the i-CAT^®^ Model 17-19 imager (Imaging Science International, Hatfield, PA, USA) operating at 1.4 mA and 120 kV, which provided a field of view 11 cm with a voxel resolution of 0.2 mm, exposure cycle of 26.9 s.^16 , 17^ The images were previously saved in a Digital Imaging and Communications in Medicine format. The CBCT images were viewed on a monitor with a 1.6 MP resolution (Dell Inc. One Dell Way, Round Rock, TX, USA) and calibrated for medical imaging, using SIMPLANT Pro 17.01 (Dentsply Implants NV, Research Campus 10, Has selt 3500, Belgium) on a computer running the Windows 7 (Microsoft, Redmond, WA, USA) system. The training on using the software and interpreting the CBCT scans was provided over several sessions; all images were reviewed, and measurements were performed by one examiner (O.D.). The inclusion criteria for the study included an age limit up to 18 years and absence of any pathological deformities in the maxilla and the mandible. The exclusion criteria were the following: 1) very narrow, blocked canals or defects such as internal and external root resorption; 2) incomplete root development as well as endodontically treated teeth and extensive caries; 3) reference points not obviously visible due to crowding and/or extensive restorations; 4) bad diagnostic quality scans (diffuse image, or distortion).

### CBCT analysis

**Figure 1:**
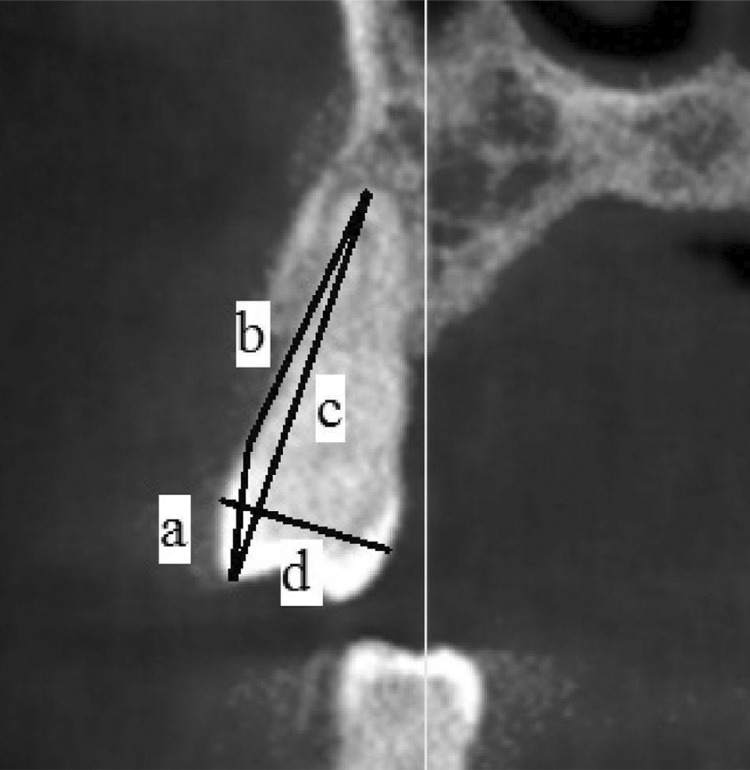
Demonstration for measuring tooth sizes. a: Crown length; b: Root length; c: Tooth length; d: Buccolingual length

The images were evaluated in the axial and cross-sectional planes. The following definitions were used for the CBCT-based measurements: a) Crown length (CL): Distance between the reference line from the buccal cementoenamel junction (CEJ) to the buccal cusp/incisal tip; b) Root length (RL): Distance between the reference line from buccal CEJ to the root apex; c) Tooth length (TL): Distance between the buccal cusp/incisal tip and the root apex; d) Buccolingual dimension (BL): Maximum buccolingual tooth length ( Figure 1 ); and e) Mesiolingual dimension:^18^ Maximum mesiodistal tooth length. Root, crown, tooth length, and buccolingual dimensions were measured on the coronal plane. The mesiodistal dimension was measured using the panoramic view in a software with all screens integrated. The number of roots and the number and type of canals were classified according to Vertucci^9^ (1974). The Vertucci’s classification is the following: a) Type I: One single canal extending from the pulp chamber; b) Type II: Two separate canals extend from the pulp chamber and merge into one canal before reaching the apex; c) Type III: One canal extends from the pulp chamber and branches into two canals, which later merge at the apical foramen; d) Type IV: Two separate canals extend from the pulp chamber to the apex; e) Type V: One canal leaves the pulp chamber and is divided into two separate canals with distinct apical foramina; f) Type VI: Two separate canals leave the pulp chamber, merge at the mid-root, and then branch again into two separate canals; g) Type VII: One canal leaves the pulp chamber and branches into two canals, which merge again at the mid-root, and then branch again into two separate canals at the apex; h) Type VIII: Three distinct canals leave the pulp chamber and extend to the apex. To test reproducibility, all measurements were performed thrice and the mean value was taken.

### Statistical analysis

Means, standard deviations and coefficients of variation for all variables were determined for all teeth by gender. The difference between means for gender-related differences was analyzed using the independent sample t test with Levene’s Test for Equality of Variances. The one-way ANOVA was used to determine a possible relation between anatomic lengths and canal morphology. The pearson’s correlation coefficient was used (H0: r=0) to determine the correlation between tooth lengths (TL, CL, RL, MD, BL) and age. All statistical analyses were performed using the SPSS software (Version 20; IBM SPSS Inc., Armonk, NY, USA).

## Results

**Figure 2:**
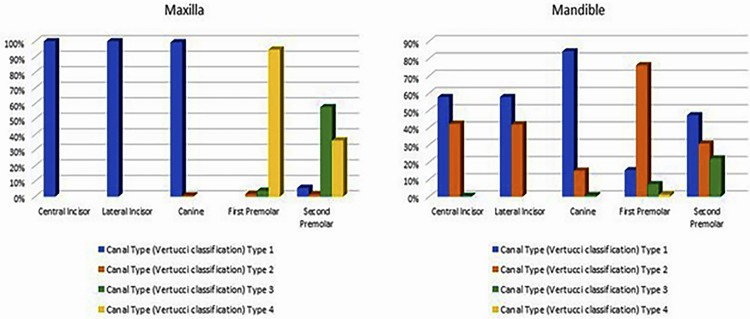
Frequency distribution of canal types in maxillary and mandibular teeth

The study sample consisted of 384 patients (n=233 females and n=151 males), providing a total of 447 CBCT scans (n=280 mandibular and n=167 maxilla) with an age average age of 52.89±17.70 years. There was no significant age difference between genders. As far as the canal topology was concerned, the Type I canal configuration was most prevalent in the maxillary anterior teeth. Most of the maxillary first premolars demonstrated the Vertucci Type IV canal system and about 60% of second premolars had the Type II variant. The mandibular incisors and canines showed a 60% and 80% predilection for Type I configuration, respectively. 76% of the mandibular first bicuspids had Type II canal system, while the canal system of mandibular second premolars showed maximal diversity, with 47% belonging to the Vertucci Type I, and about 30% and 20%, to Type II and Type III, respectively. The detailed canal topology percentage distribution for each tooth is shown in Figure 2 .

The cumulative mean of crown and root lengths, and buccolingual and mesiodistal dimensions for anterior and premolar teeth of the right and left quadrants are noted in Table 1 (maxilla) and in Table 2 (mandible). When the mean tooth anatomic lengths (TL, CL, RL, BL, MD) were compared between men and women for both the arches, almost all the anatomic measurements were higher for men than for women (p<0.05; Tables 1, 2). Additionally, there was no significant age-related differences between genders (p=0.1). Statistically significant relations between the tooth anatomic lengths and the Vertucci’s canal system type are shown in Table 3 (maxillary arch) and in Table 4 (mandibular arch). The most significant relation (p<0.05) between the anatomic size and canal topology was observed in maxillary first premolars, followed by the buccolingual dimension for mandibular canines and crown length for mandibular second bicuspids (in Tables 3 and 4). The mean tooth length was greatest with the Vertucci Type IV canal system for both maxillary right and left first premolar. The buccolingual dimension was the lowest with the Vertucci’s Type I when compared with Type III and IV ( Table 3 ). For the mandibular arch, the mean crown lengths for the second premolars were greater with the Vertucci Type III canal variant. In addition, the buccolingual dimension was also significantly larger for both mandibular right and left canine with the Type III root canal system ( Table 4 ).

**Table 1:** Comparison of tooth length measurements between male and female subjects (maxilla)

Tooth type	Variable	n	Gender	Mean ± SD	t	Sig (2 tailed)
Central Incisor	TL	57	M	23.3 ± 1.9	-3.1	0.005**
		78	F	22.3 ± 1.8		
	CL	57	M	9.7 ± 1.2	1.3	0.16
		78	F	10 ± 1.0		
	RL	57	M	13.9 ± 1.5	-2.3	0.02*
		78	F	12.9 ± 1.5		
	BL	57	M	7.62 ± 0.5	-0.2	0.84
		78	F	7.59 ± 0.6		
	MD	57	M	8.32 ± 0.3	1.44	0.15
		78	F	8.2 ± 0.6		
Lateral Incisor	TL	60	M	22.6 ± 2	-2.2	0.02*
		81	F	21.7 ± 1.7		
	CL	60	M	8.8 ± 1.1	-0.7	0.4
		81	F	8.7 ± 1.04		
	RL	60	M	14.2 ± 1.7	-2	0.03*
		81	F	13.5 ± 1.6		
	BL	60	M	7.05 ± 0.8	-0.6	0.3
		81	F	7.05 ± 0.3		
	MD	60	M	6.9 ± 0.8	0.1	0.8
		81	F	6.9 ± 0.2		
Canine	TL	62	M	26.2 ± 2.2	-3.9	0.000**
		91	F	24.8 ± 1.7		
	CL	62	M	10.1 ± 1.2	-3	0.002**
		91	F	9.5 ± 0.8		
	RL	62	M	17.8 ± 0.8	-2.1	0.02*
		91	F	16.1 ± 1.4		
	BL	62	M	8.9 ± 0.7	-2.4	0.01*
		91	F	7.9 ± 0.6		
	MD	62	M	7.8 ± 0.5	-0.7	0.4
		91	F	7.7 ± 0.6		
First Premolar	TL	43	M	21.4 ± 1.8	-3.5	0.001**
		54	F	20.1 ± 1.8		
	CL	43	M	8.1 ± 0.8	-3.2	0.001**
		54	F	7.4 ± 0.8		
	RL	43	M	13.8 ± 1.8	-2.1	0.03*
		54	F	13.0 ± 0.5		
	BL	43	M	9.6 ± 0.8	0.09	0.9
		54	F	9.55 ± 0.8		
	MD	43	M	7.3 ± 0.5	0.4	0.6
		54	F	7.2 ± 0.2		
Second Premolar	TL	30	M	21.8 ± 1.6	-2.7	0.008**
		42	F	19.8 ± 2.0		
	CL	30	M	7.7 ± 0.4	-2.7	0.009**
		42	F	7.0 ± 1.0		
	RL	30	M	14.6 ± 1.2	-2.3	0.02
		42	F	12.9 ± 0.8		
	BL	30	M	9.7 ± 0.9	-0.2	0.8
		42	F	9.7 ± 1.0		
	MD	30	M	7.2 ± 0.6	1.4	0.1
		42	F	7.1 ± 0.5		

Values presented as mean ± standard deviation in mm. Mx: Maxillary; TL: Total length; CL: Crown length; RL: Root length; BL: Buccolingual dimension; MD: Mesiodistal dimension. M: Male; F: Female. p-values significance level <0.05 using Independent t-test

**Table 2:** Comparison of the tooth length measurements between male and female subjects (mandible)

Tooth type	Variable	n	Gender	Mean ± SD	t	Sig (2 tailed)
Central Incisor	TL	104	M	21 ±1.63	-4.5	0.00**
		154	F	20 ± 1.3		
	CL	104	M	8.1 ± 1	-0.4	0.1
		154	F	8 ± 0.8		
	RL	104	M	13.7 ± 1.4	-5.4	0.000**
		154	F	12.7 ± 1.1		
	BL	104	M	6.1 ± 0.5	-2.4	0.02*
		154	F	5.3 ± 0.6		
	MD	104	M	5.5 ± 0.6	-0.0	0.9
		154	F	5.5 ± 0.65		
Lateral Incisor	TL	102	M	22.1 ± 1.8	-4.3	0.000**
		158	F	21.1 ± 1.5		
	CL	102	M	8.5 ± 1	-1.7	0.07
		158	F	8.3 ± 0.9		
	RL	102	M	14.3 ± 1	-4.6	0.000**
		158	F	13.3 ± 1		
	BL	102	M	6.5 ± 0.6	-1.6	0.09
		158	F	6.1 ± 0.6		
	MD	102	M	5.8 ± 0.6	-0.2	0.8
		158	F	5.8 ± 0.5		
Canine	TL	96	M	25 ± 2	-6.3	0.000**
		163	F	23 ± 1		
	CL	96	M	9.7 ± 1.2	-4.8	0.000**
		163	F	9.1 ± 1		
	RL	96	M	16.6 ± 1.8	-5.1	0.000**
		163	F	15.5 ± 1.6		
	BL	96	M	8.2 ± 0.7	-7.4	0.000**
		163	F	7.5 ± 0.7		
	MD	96	M	7.2 ± 0.6	-2.6	0.009**
		163	F	6.8 ± 0.5		
First Premolar	TL	78	M	22.2 ± 1.6	-4.4	0.000**
		129	F	21.1 ± 1.5		
	CL	78	M	7.9 ± 0.7	-2.6	0.008**
		129	F	7.6 ± 0.7		
	RL	78	M	15.2 ± 1.5	-3.5	0.001**
		129	F	14.3 ± 1.3		
	BL	78	M	8.2 ± 0.7	-2.9	0.004**
		129	F	7.9 ± 0.6		
	MD	78	M	7.2 ± 0.6	-1.3	0.1
		129	F	7.2 ± 0.5		
Second Premolar	TL	53	M	22 ± 1.6	-4.4	0.000**
		93	F	20 ± 1.3		
	CL	53	M	7.7 ± 0.7	-1.7	0.08
		93	F	7.5 ± 0.7		
	RL	53	M	15 ± 1.5	-4.3	0.000**
		93	F	14 ± 1.1		
	BL	53	M	8.4 ± 0.8	-1.5	0.1
		93	F	8.1 ± 0.8		
	MD	53	M	7.2 ± 0.5	-0.8	0.4
		93	F	7.2 ± 0.6		

Values presented as mean ± standard deviation in mm. Mn: mandibular; TL: Total length; CL: Crown length; RL: Root length; BL: Buccolingual dimension; MD: Mesiodistal dimension. M: Male; F: Female. p-values significance level <0.05 using Independent t-test

**Table 3:** Statistically significant relations between tooth anatomic sizes and Vertucci’s Root Canal system (maxillary arch)

Tooth Number	Variable	
14 (First Premolar)	TL (mm)	
	Vertucci Type I	20.3 ± 3.1
	Vertucci Type III	20.4 ± 2
	Vertucci Type IV	21 ± 2.1
	p value	0.03
	CL (mm)	
	Vertucci Type I	6.7 ± 2.1
	Vertucci Type III	7.8 ± 0.8
	Vertucci Type IV	7.8 ± 0.8
	p value	0.004
	BL (mm)	
	Vertucci Type I	8.9 ± 2.1
	Vertucci Type III	9.5 ± 0.7
	Vertucci Type IV	9.7 ± 0.9
	p value	0.004
24 (First Premolar)	TL (mm)	
	Vertucci Type II	19.5 ± 1.9
	Vertucci Type III	18.6 ± 3
	Vertucci Type IV	21.1 ± 2
	p value	0.04
	RL (mm)	
	Vertucci Type II	12.1
	Vertucci Type III	11.5 ± 2.6
	Vertucci Type IV	13.9 ± 1.8
	p value	0.03
	BL (mm)	
	Vertucci Type II	7.8 ± 0.9
	Vertucci Type III	9.5 ± 0.3
	Vertucci Type IV	9.5 ± 0.9
	p value	0.03
25 (Second Premolar)	BL (mm)	
	Vertucci Type I	10 ± 1.1
	Vertucci Type II	7
	Vertucci Type III	9.5 ± 0.8
	Vertucci Type IV	10.2 ± 0.9
	p value	0.001

TL: Total length; CL: Crown length; BL: Buccolingual dimension; p-values significance level <0.05 using ANOVA

**Table 4:** Statistically significant relations between tooth anatomic sizes and Vertucci’s Root Canal system (mandibular arch)

Tooth Number	Variable	
35 (Second Premolar)	CL (mm)	
	Vertucci Type I	7.4 ± 0.7
	Vertucci Type II	7.6 ± 0.7
	Vertucci Type III	7.9 ± 0.7
	p value	0.006
33 (Canine)	BL (mm)	
	Vertucci Type I	7.7 ± 0.7
	Vertucci Type II	8 ± 0.7
	Vertucci Type III	8.8
	p value	0.000
43 (Canine)	BL (mm)	
	Vertucci Type I	7.6 ± 0.7
	Vertucci Type II	8 ± 0.8
	Vertucci Type III	9.1
	p value	0.002

CL: Crown length; BL: Buccolingual dimension; p-values significance level <0.05 using ANOVA

Finally, some weak negative correlations ( Table 5 ) existed between the crown length and patient’s age for maxillary and mandibular anterior teeth and second mandibular premolar, with the crown lengths decreasing with increasing age (r=−0.2, p<0.01).

**Table 5:** Correlations between tooth anatomic lengths and age

Arch	Variable	Variable	Central Incisor	Lateral Incisor	Canine	First Premolar	Second Premolar
Maxilla	Age	TL (r)	-0.3	-0.1	0.06	0.2	0.2
		p value	0.000**	0.03**	0.44	0.04**	0.09
		CL (r)	-0.1	-0.2	-0.1	0.06	0.03
		p value	0.04**	0.003**	0.02**	0.5	0.7
		RL (r)	0.07	-0.07	0.06	0.1	0.1
		p value	0.4	0.3	0.4	0.2	0.1
		BL (r)	0.04**	-0.05	0.09	-0.1	0.03**
		p value	0.8	0.9	0.2	0.3	0.8
		MD (r)	-0.06	-0.2	-0.02**	-0.1	-0.2
		p value	0.4	0.01**	0.7	0.1	0.04**
Mandible	Age	TL (r)	-0.1	-0.04**	0.004**	-0.1	-0.08
		p value	0.08	0.4	0.9	0.02*	0.3
		CL (r)	-0.2	-0.2	-0.1	-0.1	-0.2
		p value	0.000**	0.001**	0.007**	0.08	0.003**
		RL (r)	0.03**	0.06	0.1	0.04**	0.008**
		p value	0.5	0.2	0.04*	0.55	0.9
		BL (r)	0.02**	0.02**	0.02**	-0.01**	-0.02**
		p value	0.6	0.6	0.7	0.8	0.7
		MD (r)	0.02**	-0.01**	-0.007**	-0.05	0.000**
		p value	0.7	0.7	0.9	0.4	0.9

TL: Total length; CL: Crown length; RL: Root length; BL: Buccolingual dimension; MD: Mesiodistal dimension. p-values significance level <0.05 using Pearson correlation test

## Discussion

To account for the possible root canal variations, this study was designed to expand the understanding of the anatomic morphology of the maxillary and mandibular anterior and premolar teeth.^11^ Adequate knowledge about the internal teeth anatomy is extremely vital for a successful endodontic therapy. Due to the two-dimensional nature of the periapical radiographs, the detection of anatomical complexities substantiates the use of more detailed digital radiography. This current study provides a comprehensive report on the morphology of root canals for maxillary and mandibular anterior teeth and premolars, the relation to crown and root sizes, and bucco-lingual and mesio-distal tooth dimensions using CBCT analysis.

According to previous studies,^5 , 15^ the maxillary central and lateral incisors assessed in our study population were single-rooted and predominantly belonging to the Vertucci canal Type I, for a total of 167 maxillary scans studied. Çalişkan, et al.^5^ (1995) noted that 93.48% of maxillary canines had Type I canal configuration in a Turkish population. In another study, Sert and Bayirli^19^ (2004) reported that the root canal configurations for maxillary canines were 91% Type I, 3% Type II, 4% Type III, and 2% Type IV. Likewise, in this study, 99% of the maxillary canines demonstrated type I configuration with approximately 1% Type II. The root canal morphology of mandibular incisors is reported to vary greatly, and the difference in prevalence is affected by factors such as sample size, examination methods, and ethnic diversity.^6 , 8 , 10^ Studies using staining and clearing techniques^20 , 21^ have reported that the prevalence of a single canal in mandibular incisors is around 65.6%, and 36.25% with two canals. This is consistent with the results of this study. In a total of 280 mandibular scans, 58% of the lower incisors have the Vertucci Type I followed by 41.5% Type II, and 0.5% Type III configuration. The mandibular canines showed a greater predilection for the Type I (85%) canal configuration, followed by the Type II (14%), and the Type III (1%) variants, with the buccolingual dimension increasing with these rare analogs. It is important to always remember that observing a sudden narrowing or attenuation in the canal path radiographically may indicate the possibility of canal bifurcation.

Several reports note the variances in the morphology of canal premolars among different ethnic groups.^22 - 24^ A study found black patients have a higher number (32.8%) of extra canals in mandibular premolars than white patients (13.8%).^22^ Walker determined that Asians have different canal configuration percentages in maxillary and mandibular premolars than those reported in Caucasians and Africans.^23^ Besides the conventional Type IV (94%) canal system for the maxillary first premolars, it was found an average of 4% and 2% of these premolars with the Type III and Type II variants, respectively. Additionally, the total tooth length, crown length, root length and buccolingual dimension was significantly lower with these two variants compared with the conventional Type IV canal maxillary first premolar. In previous studies, changes in crown sizes with bifid versus singular canal teeth were observed.^25 , 26^ In a full-mouth radiographic survey, Serman and Hasselgren^27^ (1992) found 15.7% of mandibular first premolars and 7% of mandibular second premolars had a divided canal. In our study, 75% of the mandibular first premolars exhibited the Type II canal anatomy, while mandibular second premolars appeared to manifest maximal diversity in canal configuration terms with 46%, 30% and 22% of these teeth belonging to the Vertucci’s Type I, Type II and Type III category, respectively. The frequency estimates of lower first premolars that possess more than one canal was found to be 21.9% in a Spanish population, 37.5% in a Chinese study; re-emphasizing the ethnicity connection to these anatomic differences.^24 , 28^ Likewise, we did find single canal mandibular first premolars to be predominant. Because of geographic differences, it was observed far less (~2%) two canal variants; although it was noticed a high single canal variant Type II proportion: two separate canals leave pulp chamber and join short of the apex to form one canal. The crown lengths for mandibular second premolars appeared to be significantly greater with the Type III classification (one canal leaves pulp chamber and are divided into two canals, which finally merge into one). Clinicians must always consider the presence of a divided canal in such teeth and modify access cavity preparations accordingly, such as further removal of the pulp chamber roof of the lingual part to access the canal to avoid the likelihood of pulpal necrosis due to partial endodontic treatment.

Gender has been invariably reported as an important factor to be considered in the preoperative evaluation of the canal morphology for root canal treatment.^19^ In this study, all the anatomic measurements were higher in males than in females. The pulp cavity generally decreases in size as an individual ages^4^ with non-uniform dentine and cementum formation occurring throughout life and is more rapid on the roof and floor than on the pulp chamber walls of the posterior teeth. These calcifications result in a flattened pulp chamber. In accordance with previous studies,^4^ a weak negative correlation was noticed between crown lengths and increasing age. This study did not examine the correlation between tooth lengths and persons’ stature.

### Clinical relevance

The data obtained in this study can be used as a reference for evaluating CBCT based measurements of: tooth, crown and, root lengths, as well as bucco-lingual/mesio-distal dimensions of anterior and premolar teeth. Considering the apparent relationship between the tooth anatomic lengths and the root canal variants, meticulous radiographic interpretation, proper access preparation, and a detailed tooth exploration with age and gender consideration are essential prerequisites for a successful treatment outcome.

### Limitations and future directions

The proportion of women in this study was higher than that of men (60% versus 40%). Additionally, subtle incongruities were observed in the findings of this study when compared with other studies, due to differences in geographic location (ethnic and genetic factors) and assessment methods. Future studies using CBCT-based measurements with larger sample size can help augment these findings.

## Conclusions

In summary, maxillary and mandibular anterior teeth prevalence is higher for Type I canal configuration. Anatomic lengths (crown and root length, buccolingual and mesiodistal dimension) had the strongest influence on the root canal configuration of maxillary first premolars. Additionally, mandibular second premolars showed maximal diversity concerning the type of canal classification and a significant correlation with their crown lengths.
